# Photo Quiz: Appendiceal agony and hepatic havoc: exploring infection in the immunocompromised

**DOI:** 10.1128/jcm.00562-24

**Published:** 2025-04-09

**Authors:** Bryce Bobb, Tristan Jones, Bryce Hatfield, David Friedel, William Koch, Colin Thibodeau, Christopher Doern, Alexandra L. Bryson

**Affiliations:** 1 Department of Pathology, Virginia Commonwealth University Health System 543105 , Richmond, Virginia, USA; 2 Department of Pediatrics Division of Infectious Diseases, Virginia Commonwealth University Health System 466504 , Richmond, Virginia, USA; Mayo Clinic Minnesota, Rochester, Minnesota, USA

**Keywords:** fungi, *Magnusiomyces*, disseminated, gastrointestinal infection, MALDI, hematologic malignancies

## PHOTO QUIZ

A 3-year-old girl with a history of high-risk B-cell acute lymphoblastic leukemia in consolidation phase of chemotherapy presented to the hospital with fever in the setting of profound neutropenia. A complete blood count revealed absolute leukopenia of 200 × 10^6^/L white blood cells, and a comprehensive metabolic panel revealed mildly elevated hepatic enzymes. The serum β-1,3-D-glucan level (Fungitell, KSL Diagnostics Inc., Buffalo, NY) was 412 pg/mL. Computed tomography imaging obtained with intravenous contrast showed appendicitis without other bowel enhancement, innumerous hypodensities in the liver and kidneys concerning for micro-abscesses, and several 2–3 mm pulmonary nodules.

**Fig 1:**
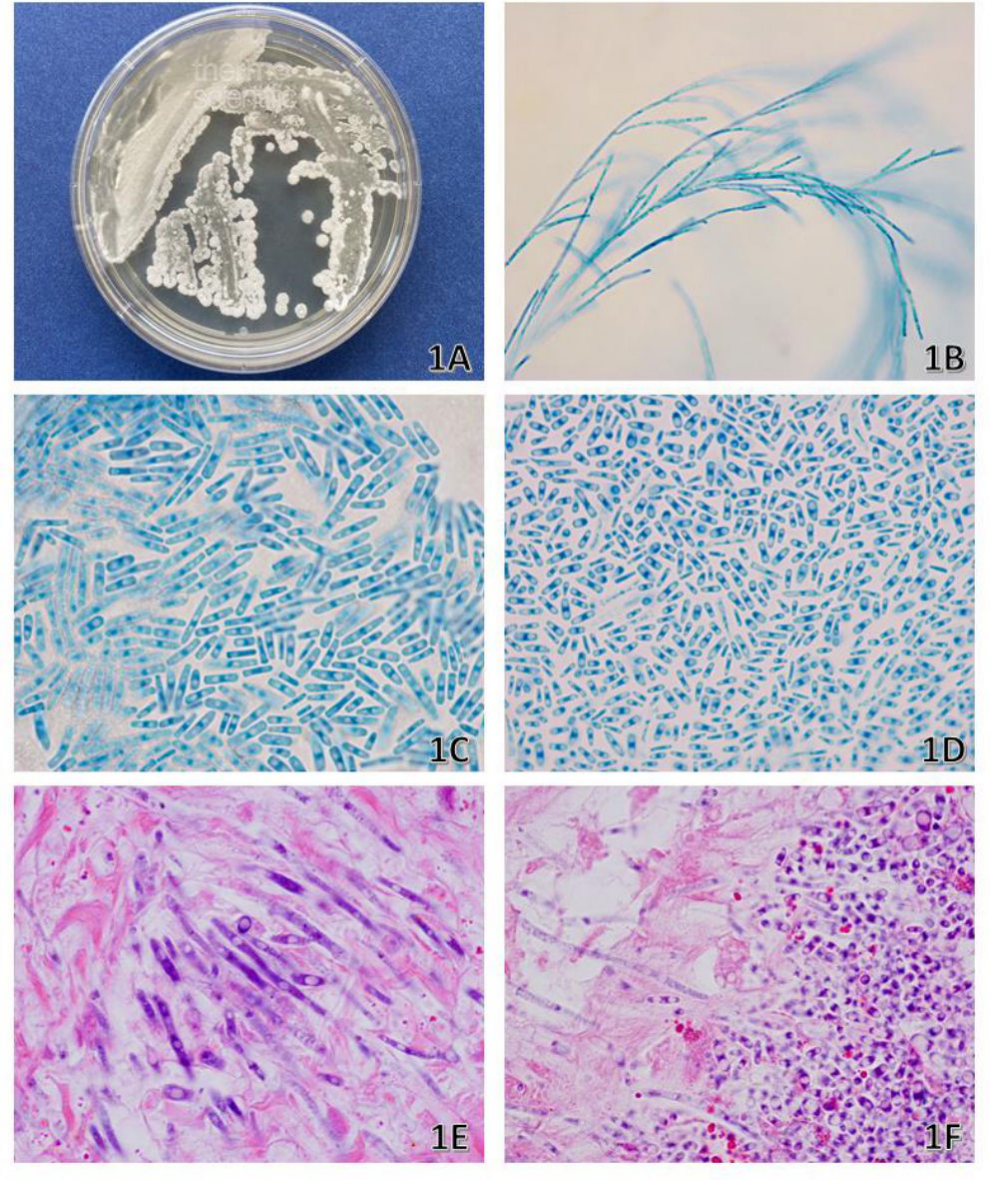
(A) Culture growth at 6 days on Sabouraud dextrose agar. (B) Lactophenol cotton blue (LPCB) tape prep: 400x magnification showing branched hyphae. (C and D) LPCB tape prep: 1,000x magnification showing rectangular arthroconidia of variable size. (E and F) Hematoxylin and eosin stain from the appendix showing fecalith and appendiceal tissue: 1,000x magnification.

The patient underwent exploratory laparotomy for appendectomy and sampling of hepatic abscesses. The appendix histopathology yielded acute inflammation and an organism, which was visualized on hematoxylin and eosin stain (Fig. 1E and F). Peritoneal fluid, liver tissue, and peritoneal tissue, obtained intraoperatively, were submitted for fungal and bacterial culture. The fungal culture grew white colonies on inhibitory mold and brain heart infusion agar (Remel, Lenexa, KS), while a Sabouraud dextrose agar, Emmons (Thermo Fisher Scientific, Waltham, MA) agar, grew dry white colonies (Fig. 1A). On day 3 of incubation, the bacterial culture grew dry white colonies on blood agar, chocolate agar, Columbia NA w/5% sheep blood agar, and MacConkey agar (Remel, Lenexa, KS), while the anaerobic media were negative for growth. Lactophenol cotton blue tape prep and slide culture prepared from potato flake agar (Remel, Lenexa, KS) subcultures were performed (Fig. 1B through D). The organism was identified by matrix-assisted laser desorption/ionization time-of-flight mass spectrometry and Sanger sequencing.

## PHOTO QUIZ ANSWER

This is a case of *Magnusiomyces* spp. (alternatively known as *Saprochaete* spp. and *Geotrichum clavatum*). Matrix-assisted laser desorption/ionization time of flight mass spectrometry (MALDI-TOF) identified the isolate as *Saprochaete clavatus* (score: 1.71) (Bruker; MALDI Biotyper; MBT smart; BDAL version 9), which per our laboratory developed MALDI-TOF procedure is a high-enough score to report to the genus level, but not the species level. Internal transcriped spacer (ITS) sequencing demonstrated a 99.74% identity to several *Magnusiomyces* species including *Magnusiomyces clavatus* and *Magnusiomyces capitatus* (SMART Gene’s, Integrated Database Network System Fungi References, accessions: OW988511 and OW984254). *Magnusiomyces* produces true hyphae that disarticulate into arthroconidia of variable sizes (1). This environmental organism is found worldwide and has been identified in wood, soil, and animals and as part of normal microbiota of human skin (2). *Magnusiomyces* is an uncommon cause of human infection but when reported has mainly been described in disseminated infections, associated with deep organ involvement in patients with profound neutropenia due to chemotherapy and hematologic malignancy. It can cause superficial infections of mucocutaneous surfaces, presenting as oral thrush, esophagitis, or skin lesions (2). A retrospective study reported β-1-3-D-glucan was detected in 15/23 invasive *Magnusiomyces* infections with 65% sensitivity and 96% specificity (3, 4). Treatment of *Magnusiomyces* infections remains challenging due to limited antifungal options and intrinsic resistance to echinocandins (2). Management typically involves systemic antifungal therapy with agents such as amphotericin B, voriconazole, posaconazole, or isavuconazole. However, treatment outcomes are often poor, emphasizing the importance of early detection, prompt initiation of therapy, and aggressive management of underlying predisposing conditions (2).

Although there are no official breakpoints, susceptibility testing was performed by broth microdilution according to the methods published in the CLSI M27 document 3rd edition (Fungus Testing Laboratory, UT Health San Antonio), which revealed the following minimal inhibitory concentrations: isavuconazole (0.25 µg/mL), posaconazole (0.25 µg/mL), and voriconazole (0.06 µg/mL). The patient was treated with liposomal amphotericin-B for 2 weeks and then transitioned to isavuconazole for prolonged therapy. The serum β-(1, 3)-D-glucan level decreased to 126 pg/mL at 4 weeks and 90 pg/mL at 8 weeks. She has since resumed chemotherapy, and repeat imaging while on antifungal therapy showed a decrease in the number of pulmonary nodules but no improvement in the liver abscesses.
